# Passive sampler phases for pesticides: evaluation of AttractSPE™ SDB-RPS and HLB versus Empore™ SDB-RPS

**DOI:** 10.1007/s11356-020-12109-9

**Published:** 2021-01-12

**Authors:** Benjamin Becker, Christian Kochleus, Denise Spira, Christel Möhlenkamp, Julia Bachtin, Stefan Meinecke, Etiënne L. M. Vermeirssen

**Affiliations:** 1 German Federal Institute of Hydrology (BfG), Am Mainzer Tor 1, 56068 Koblenz, Germany; 2 German Environment Agency (UBA), Schichauweg 58, 12307 Berlin, Germany; 3 Swiss Centre for Applied Ecotoxicology, Überlandstraße 133, Dübendorf, 8600 Switzerland

**Keywords:** Chemcatcher, Passive sampling, Stream channel, SDB-RPS, Sampling rates, Integrative ratio

## Abstract

In this study, three different passive sampling receiving phases were evaluated, with a main focus on the comparability of established styrene-divinylbenzene reversed phase sulfonated (SDB-RPS) sampling phase from Empore™ (E-RPS) and novel AttractSPE™ (A-RPS). Furthermore, AttractSPE™ hydrophilic-lipophilic balance (HLB) disks were tested. To support sampling phase selection for ongoing monitoring needs, it is important to have information on the characteristics of alternative phases. Three sets of passive samplers (days 1–7, days 8–14, and days 1–14) were exposed to a continuously exchanged mixture of creek and rainwater in a stream channel system under controlled conditions. The system was spiked with nine pesticides in two peak scenarios, with log *K*_OW_ values ranging from approx. − 1 to 5. Three analytes were continuously spiked at a low concentration. All three sampling phases turned out to be suitable for the chosen analytes, and, in general, uptake rates were similar for all three materials, particularly for SDB-RPS phases. Exceptions concerned bentazon, where E-RPS sampled less than 20% compared with the other phases, and nicosulfuron, where HLB sampled noticeably more than both SDB-RPS phases. All three phases will work for environmental monitoring. They are very similar, but differences indicate one cannot just use literature calibration data and transfer these from one SDB phase to another, though for most compounds, it may work fine.

**Supplementary Information:**

The online version contains supplementary material available at 10.1007/s11356-020-12109-9.

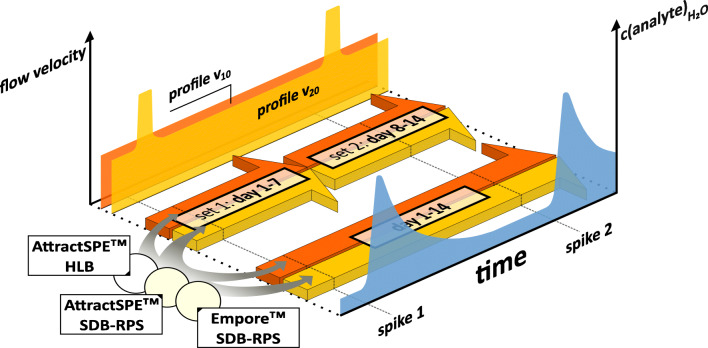

## Introduction

Passive sampling is considered to be an important tool to further improve water monitoring. Therefore, it is a recommended approach for future application in the EU Water Framework Directive (EU-WFD) (Brack et al. 2017). Several aspects make passive sampling an attractive method of choice for monitoring chemicals in the water phase. First, it enriches compounds in situ so that extraction of (large) water sample volumes prior to analysis is not necessary. This is particularly useful for hydrophobic organic compounds such as polychlorinated biphenyls (Smedes et al. 2010) or some insecticides (Moschet et al. 2015) and allows for low detection limits. Second, passive sampling provides a time-weighted average concentration. Obtaining such a sample with conventional methods is either laborious (high frequency grab sampling) or costly (i.e., requiring automated samplers). Third, environmental matrix is reduced due to selective sampling of target analytes in situ and, therefore, results in relatively clean extracts (Smedes et al. 2010). These advantages make passive sampling a well-suited method to monitor pesticides, which are the analytes of interest in this study. Pesticides undergo high fluctuations due to different usages in agriculture, industry, and domestically. Furthermore, they greatly vary in their mobility and persistence. Grab sampling is an inadequate method to reliably detect these varying concentrations (Taylor et al. 2020). For this reason, passive sampling, with advantages of time-weighted average concentrations and low detection limits, is very beneficial for sampling of event-based contaminations. Despite these and other advantages (Booij et al. 2016), implementing passive sampling also has its challenges. Accurate calculation of time-weighted concentrations requires careful calibration and ideally integrates a method to correct for sampler performance in the field (Booij et al. 2016). The passive sampling method currently also has limitations concerning compliance monitoring with respect to environmental quality standards (EQS) as implemented within the EU-WFD (European Commission 2000). These threshold values are based on total concentration (*c*_total_) (Booij et al. 2016), whereas passive sampling determines the freely dissolved concentration (*c*_free_). Notwithstanding these limitations, the further implementation of passive sampling methods is likely to improve monitoring methods significantly. Miège et al. (2015) and many other studies already showed the potential of passive sampling in monitoring of hydrophobic (Booij et al. 2016; Smedes and Booij 2012) and hydrophilic analytes (Charriau et al. 2016; Lissalde et al. 2016). Also, for specific aims like pesticide monitoring, good correlations between passive sampling and integrated active sampling were shown by Ahrens et al. (2015). To further advance the use of passive sampling in water monitoring, standardization of methods and materials is much needed (Booij et al. 2016). Therefore, uniform and comparable products with a secure production line are needed to advance passive samplers to a robust monitoring method. Without standardized samplers, a comparison between different passive sampling studies is difficult. This is shown by Taylor et al. (2019), who explain many advantages off passive sampling for hydrophobic organic compounds as it overcomes shortcomings of biota and grab sampling. However, they stress the importance of standardized methods and sampler design to enhance acceptance and comparability of the passive sampling method. Furthermore, they highlight the importance of certified reference material to guarantee reliable and accepted water-polymer partition coefficients as well as polymer diffusion coefficients for passive sampler sorbent phases.

The project PASTraMi aimed at evaluating passive samplers for monitoring a defined set of substances (Table 1). To cover this range of compounds with a single sampler type, SDB-RPS disks, which provide a sampling phase typically used for solid phase extraction, were selected. One reason for selecting SDB-RPS disks over another popular format namely POCIS (two membranes enclosing solid phase extraction sorbent) was that SDB-RPS disks can be used both with and without a protective and diffusion limiting membrane. Since common membranes are known to strongly sorb more hydrophobic compounds (Alvarez 1999; Endo and Matsuura 2018; Tran et al. 2007), it must be checked whether it is suitable to use a membrane for the particular compounds of interest before SDB-RPS disks can be used in practice. This study was conducted solely without membranes since preliminary tests showed that some analytes targeted in the PASTraMi project were completely retained by the membrane (see Fig. S1). Another advantage of a sampler without a membrane is that higher sampling rates can be achieved per unit of area (Schäfer et al. 2008). This is particularly useful for the shorter sampling windows set out for PASTraMi, namely 1- to 2-week periods.

**Table 1:** Properties of target analytes (*n* = 9) spiked during two peak concentration events and to provide constant background concentrations (*n* = 3). Metazachlor ethane sulfonic acid (ESA) was not spiked but analyzed as the major degradation product of metazachlor. Octanol-water partition coefficients (log *K*_OW_) were taken from US EPA (2017) unless indicated otherwise. Octanol-water distributions coefficients at pH = 7 (log *D*_ow_) are listed for (partly) ionized compounds

Type	Substance	CAS	Log *K*_OW_	Peak concentration (ng L^−1^)	Background concentration (ng L^−1^)
Herbicide	Bentazon	25057-89-0	− 0.93 (log D_OW_) ^b^	215	21.5
	Flufenacet	142459-58-3	3.20	215	–
	Metazachlor	67129-08-2	2.13	215	21.5
	Metazachlor ESA	172960-62-2	− 0.74 (log *D*_OW_)	–	–
	Nicosulfuron	111991-09-4	0.01 (log *D*_OW_)^c^	215	21.5
	Pendimethalin	40487-42-1	5.20	215	–
	Terbuthylazine	5915-41-3	3.40	215	–
Fungicide	Propiconazole	60207-90-1	3.72	215	–
Insecticide	Imidacloprid	105827-78-9	0.57	215	–
	Thiacloprid	111988-49-9	2.33^a^	215	–

p*K*_a_ (bentazon): 3.73 (source: http://www.t3db.ca); p*K*_a_ (metazachlor ESA): − 0.98 (https://chemicalize.com); p*K*_a_ (nicosulfuron): 4.6 (https://pubchem.ncbi.nlm.nih.gov)

^a^Calculated from molecular fragments.

^b^Calculated from log *K*_OW_ and p*K*_a_, assuming that only the non-ionized species partitions into octanol

^c^US EPA (2017)

Until 2018, Empore™ SDB-RPS disks produced by 3M™ (St. Paul, MN, USA) were broadly used solid phase extraction (SPE) disks. In general, they were used for purification and pre-concentration of analytes from aqueous samples. They were designed for efficient extraction, even from small sample volumes, and to reduce matrix effects while concentrating the sample. Hence, they allowed for more efficient analyses. These properties made SDB-RPS disks well suited for the use as passive sampler. Furthermore, they were widely used as receiving phase by the scientific community for Chemcatcher®. This phase was particularly suited for hydrophilic and hydrophobic compounds with log *K*_OW_ values between − 2 and 6 (Charriau et al. 2016). As the production of this phase was halted in 2018, uncertainty arose about the continuation of this sampling phase. Thus, there was significant and worldwide interest in a suitable replacement or alternative material. During the course of this study, production of this phase was transferred to another company, securing a longer-term availability. Still, an evaluation of suitable alternatives will expand application opportunities and secure long-term monitoring strategies and methods. Long-term availability of passive sampler phases is of great importance to prevent disruption in passive sampling research since the process was hampered already in the past by a production stop of another much used and well calibrated sampling phase (i.e., AlteSil™ silicone). In autumn 2018, Affinisep (Petit Couronne, France) started offering comparable products to Empore™, namely their AttractSPE™ line. Therefore, the aim of this study is to evaluate sampling properties of AttractSPE™ SDB-RPS disks in comparison to Empore™ SDB-RPS disks. Alongside, SDB-RPS disks also AttractSPE™ hydrophilic-lipophilic balance (HLB) have become available as a sampling phase. As an HLB phase (Atlantic® HLB SPE Disk) has been successfully applied for monitoring of emerging pollutants in South Africa (Rimayi et al. 2019) and monitoring metaldehyde in surface water (Castle et al. 2018), we included the Attract HLB platform in our study along both SDB-RPS phases as further alternative. The main goal was to explore long-term options with respect to passive sampler phases and develop knowledge on their sampling properties and comparability for ongoing and future monitoring studies. Therefore, we tested three phases under identical conditions (same housing, same flow, same temperature, same matrix); hence, we expected transport of compounds to the disk surface to be the same for all configurations. However, differences between sorbent phases, sorbent capacity, and porosity may still lead to differences in uptake.

As our usual monitoring activities take place in rivers, this evaluation was performed using a system as close to monitoring applications as possible (Vermeirssen et al. 2008). A relevant set of pesticides was tested covering a broad log *K*_OW_ range (est. − 1.15 to 4.82; values calculated by US Environmental Protection Agency’s Estimation Programs Interface (EPI Suite) v4.1; Table 1) to robustly evaluate the selectivity of the tested sampling phases. To remain close to future monitoring applications, ambient river water mixed with rainwater was used as a matrix for the uptake experiment.

## Materials and methods

### Three receiving phases, conditioning, and sampler housings

Two types of SDB-RPS disks were used, one from 3M™’s Empore™ (E-RPS) product line and the alternative was from Affinisep’s AttractSPE™ (A-RPS) product line. Furthermore, HLB divinylbenzene disks with hydrophilic moieties were also selected from Affinisep’s AttractSPE™ line. Each type of sampling disks measured 47 mm in diameter.

All disks were conditioned in batches according to Scheithauer (2015). Each type of disk was put into separate beakers filled with 20 mL of methanol per disk. After gently shaking for 30 min, methanol was decanted and solvent remains were removed by adding 20 mL of double-distilled water per disk. Thereafter, the water was decanted and fresh water (20 mL per disk) was added followed by gentle shaking for 30 min. Finally, disks were mounted into stainless steel (SST) housings (2 mm thickness) with a single-sided opening of 40 mm in diameter (Fig. S2) (Vermeirssen et al. 2012). Samplers were wrapped in aluminum foil and stored in double-distilled water until use.

### Target analytes and standards

Nine pesticides were selected, spiked to the water in the channel system and analyzed in sampler extracts. These compounds included six herbicides (bentazon, flufenacet, metazachlor, nicosulfuron, pendimethalin, terbuthylazine), one fungicide (propiconazole), and two insecticides (imidacloprid, thiacloprid) (Table 1). Furthermore, one degradation product of metazachlor, metazachlor ethane sulfonic acid (ESA), was examined, but not actively added to the system. Deuterated standards of these substances were used for identification and quantification.

### Stream channels

**Fig. 1:**
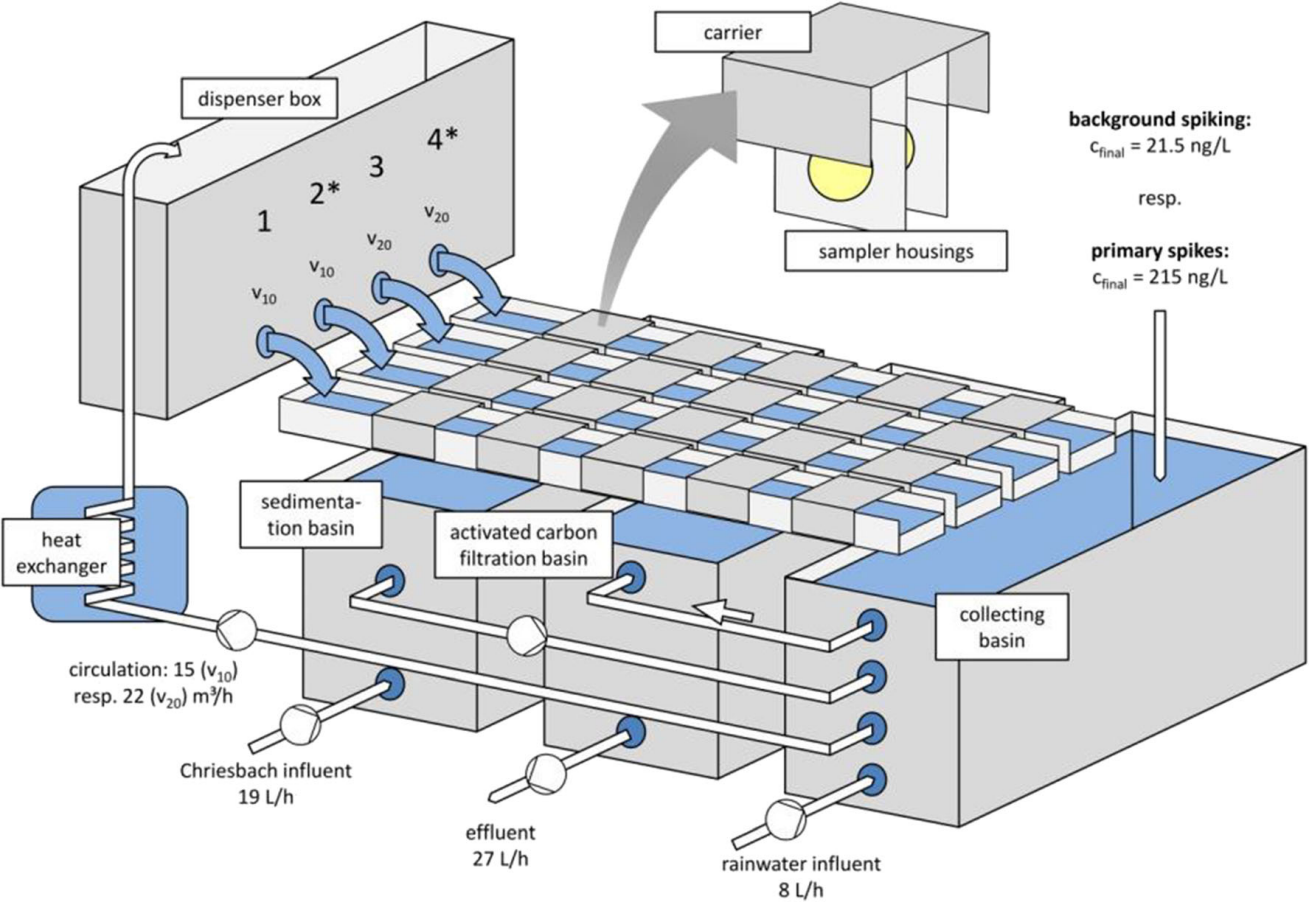
Scheme of the experimental setup. Four channels were fed with a mixture of 70% river water and 30% rain water. During the experiment, a constant background spiking was performed resulting in a 21.5-ng L^−1^ nominal water concentration of bentazon, metazachlor, and nicosulfuron. Each channel was equipped with two carriers for Empore™ SDB-RPS disks, two for AttractSPE™ SDB-RPS disks and one for AttractSPE™ HLB disks, each carrying two samplers. General flow velocity was *v*_10_ = 10 cm s^−1^ but was increased in two channels (3 and 4) to *v*_20_ = 20 cm s^−1^ during two 8-h periods following the primary spikes of nine compounds (Table 1). During these periods, water exchange and, consequently, background spiking were interrupted. After 7 days, samplers from channels 2 and 4 were exchanged (marked with asterisk)

The channel system (Fig. 1; also see Fig. S3) was operated with a volume of approximately 550 L and supplied with a dynamic mixture of 70% water from the small Swiss creek Chriesbach (47.404472° N, 8.609405° E) and 30% collected rainwater (rainwater was added to prevent calcification) with a water exchange rate of 27 L h^−1^. Water temperature (HOBO UA-002-08 8K Pendant®, Onset Computer Corporation, Bourne, MA, USA) and pH (pHD-s SC, Hach Lange GmbH, Düsseldorf, Germany) of Chriesbach influent and the circulating system were automatically logged. During the experiment, water temperature ranged between 14 and 18 °C and pH between 8.2 and 8.4 (see Fig. S4).

Input of particulate matter to the channel system was reduced from creek water by means of a sedimentation basin. Creek water and rainwater were mixed in the collecting basin at the rear end of the channels. From there, water was pumped through a heat exchanger—to maintain the water temperature close to the ambient temperature of the Chriesbach—to a dispenser box with four outlets. From this dispenser box, four identical channels (*l* = 265 cm, *w* = 10 cm, *h*_water_ = 10 cm each) were fed with water. Flow rates were controlled by changing the diameter of the outlet holes of the dispenser box feeding each channel. Flow was regulated to two different velocities *v*_10_ = 10 cm s^−1^ and *v*_20_ = 20 cm s^−1^. To compensate for the addition of fresh water (i.e., 27 L h^−1^), the same amount of water flowed out of the channel system by passive overflow. This water passed through an activated carbon filter before draining to the sewerage system.

SST housings were attached in pairs to metal carriers, which traversed the channels. Per channel, two carriers were used for each type of SDB-RPS sampler and, due to limited space in the channels and a clear focus on SDB-RPS disks, only one carrier for HLB samplers. This resulted in *n* = 4 for SDB-RPS and *n* = 2 for HLB. To avoid systematic variations in certain local conditions and ensure a homogeneous exposure for all samplers, a daily intra- and inter-channel exchange of the carriers took place.

### Experimental setup

The experiment was conducted over 14 days. Before exposure of samplers in the channels, a constant background concentration of 21.5 ng L^−1^ of bentazon, metazachlor, and nicosulfuron was applied. Flow velocity was set to *v*_10_ = 10 cm s^−1^ in all four channels. On day 2, flow rates were increased to *v*_20_ = 20 cm s^−1^ in channels 3 and 4 and a final concentration of 215 ng L^−1^ of all analytes (except for the transformation product metazachlor ESA; Table 1) was applied to the collecting basin. For the following 8 h, both water exchange and background spiking were paused to maintain this high concentration. Thereafter, flow in *v*_20_ channels was set back to 10 cm s^−1^. After 7 days, all samplers, which were originally placed in channels 2 (*v*_10_) and 4 (*v*_20_), were replaced, samplers in the other channels remained until the end of the experiment. An identical peak was applied on day 12 under the same conditions.

Water samples were taken once per day from the collecting basin and every 2 h in one *v*_10_ and one *v*_20_ channel during peak applications respectively and immediately filtered using 0.45-μm PTFE filters. Furthermore, (blank) water samples were taken from Chriesbach and rain water. Moreover, all three kinds of passive samplers were exposed to Chriesbach water, rainwater, and double-distilled water as negative controls. The course of the water concentrations in the channels is illustrated in Fig. S5.

Flow velocity was measured daily approximately 5 cm in front of every single sampler (MiniAir2, Schiltknecht, Gossau, Switzerland).

### Preparation of passive sampler extracts

All three types of disks were extracted identically. Immediately after sampling, each disk was put into 6 mL acetone and stored at 4 °C until further treatment. After shaking for 60 min at room temperature, the supernatant was collected and two more extraction steps were carried out with 6 mL methanol and 6 mL acetone. All three fractions were pooled, and the volume of solvent was set to a total of 4 mL using nitrogen stream evaporation in a 40 °C water bath.

### Chemical analysis

From each passive sampler extract, 100 μL filtrated (0.45 μm, PTFE) extract were used for analysis. 5 μL isotope labelled internal standard and 895 μL LC-MS grade water were added resulting in a 1:10 dilution. All target analytes are listed in Table 1. Samples were analyzed using a 1260 Infinity HPLC (Agilent Technologies, Santa Clara, CA, USA) coupled with QTrap 4500 tandem mass spectrometer (ABSciex, Farmingham, MA, USA) with a Kinetex® C18 column (Ø_I_ = 2.1 mm; *L* = 100 mm) with precolumn (Ø_I_ = 2.1 mm; *L* = 2 mm; Phenomenex SecurityGuard™ Ultra Cartridge UHPLC C18). The mobile phase consisted of 4 mM ammonium acetate in water (A) and methanol (B). The gradient started with 90% A and 10% B for 2 min, followed by 6 min 60% A and 40% B, and ended with 5 min 20% A and 80% B. The column was re-equilibrated with 90% A and 10% B for 7 min before each analysis. The flow rate was set to 400 μL min^−1^. ESI positive and ESI negative measurements were combined in one run. Detailed information on the method can be found in Fig. S6.

## Results and discussion

### Minor analyte uptake differences across sampling phases

**Fig. 2:**
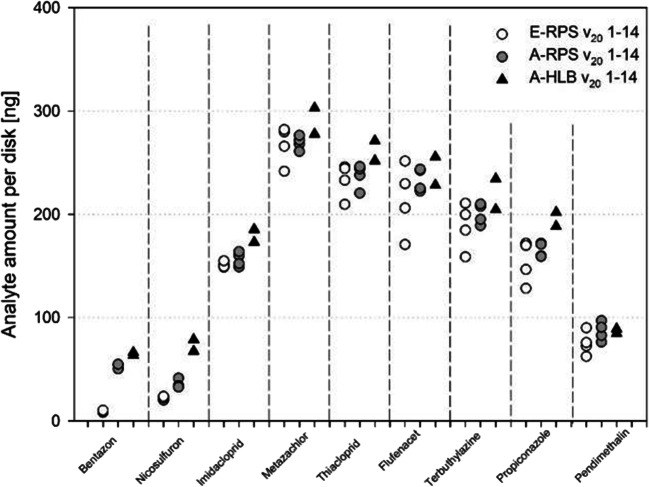
Analyte concentrations in nanograms per disk after 14 days of exposure at *v*_20_ during analyte impulses in the stream channel spiked with nine compounds (substances are sorted by log *K*_OW_). Empore™ SDB-RPS and AttractSPE™ SDB-RPS disks were sampled in four replicates. AttractSPE™ HLB disks were sampled in duplicates

Figure 2 and Fig. S7 show the sampled amounts of analyte mass per disk. In Fig. 2, the uptake after 2 weeks is shown for *v*_20_; in Fig. S7, uptake is shown for each individual week and again *v*_20_. Differences between sampling phases are small for most analytes, except for bentazon and nicosulfuron and to some degree propiconazole. Nicosulfuron and propiconazole show slightly higher amounts in the HLB sorbent phase compared with the SDB-RPS phases, possibly indicating a higher partitioning coefficient towards HLB compared with SDB-RPS. Even including these exceptions, data sets of the three phases were highly correlated with the correlation factors *r* exceeding 0.97 (see Fig. S8).

The biggest differences between the phases occurred for bentazon, which shows a five to six times higher amount in the Affinisep phases. Particularly, the difference between the two SDB-RPS phases is intriguing, as all other substances are sampled fairly equal by both SDB-RPS type phases (with minor exceptions for nicosulfuron and propiconazole). The observation regarding the markedly different behavior of both SDB-RPS types with respect to bentazon is robust. The pattern seen for bentazon in Fig. 2 was also observed in the other scenarios tested during this study (Fig. S7; data for *v*_10_ are shown in Fig. S9). There is no hypothesis to explain this different behavior of bentazon on both SDB-RPS phases. SDB-RPS disks produced by 3M™ and Affinisep have a different texture (Affinisep disks are stiffer). However, this and possibly other disk properties (SDB-polymer loading as a percentage of the disk, roughness of the disk surface, carrier for SDB-polymer) do apparently not affect uptake for the other compounds. Furthermore, similar uptake is expected, as water boundary layer conditions were the same for all three configurations. Even though both phases are made up of SDB-RPS, there may be subtle differences in the SDB-RPS material or SDB-RPS carrier formulation that affect specific compounds, possibly compounds with low log *K*_OW_ values such as bentazon or compounds that are charged. At the pH conditions in this study, bentazon is charged to almost 99%. Supposing that the neutral fraction is sampled equally by both, E- and A-RPS, A-RPS (or A-RPS/HLB carrier material) only needs to sample 5% of the charged form to generate a 5-fold difference between E- and A-RPS. To further evaluate if bentazon is a sole exception requires testing of a larger set of compounds and particularly compounds with lower log K_OW_ values or compounds that occur in a charged form. As mentioned earlier, the PASTraMi compound set was limited, because the focus of the project is to study uncertainties in the use of passive samplers and possible limitations in event-based pollution and only covered two compounds that are charged at pH 8.

As mentioned in the methods, capacity limitations of the channels restricted the possibilities for testing more than two HLB disk replicates (or more HLB-phases). Moreover, as an analytical issue with one HLB replicate for the 2-week *v*_10_ treatment was encountered (see Fig. S9-1), this somewhat complicates the interpretation of HLB disk results. However, as the study covers several sub experiments (1- and 2-week slots as well as the different flow regimes), the assessment that HLB behaves largely similar to SDB-RPS is robust (correlation A-RPS and A-HLB see in Fig. S10).

### A brief increase in flow rate leads to higher uptake

**Fig. 3:**
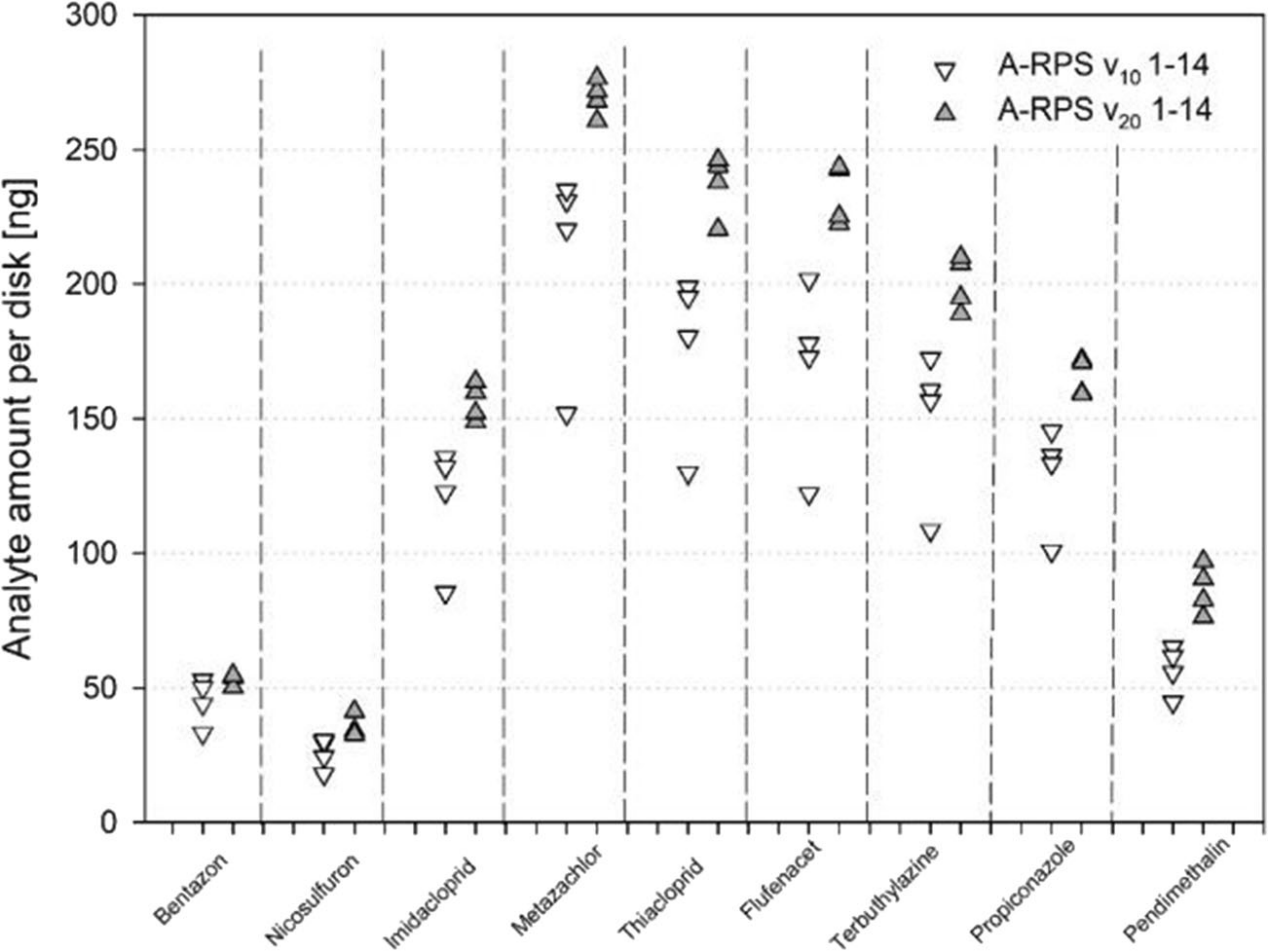
Analyte concentrations in nanograms per disk after 14 days of exposure in the stream channel at *v*_10_ (left) and at *v*_20_ (right). Bentazon, metazachlor, and nicosulfuron were added as a continuous background concentration of 21.5 ng L^−1^. Additionally, a 215-ng L^−1^ peak of all analytes was applied for 8 h on day 2 and day 12. Empore™ SDB-RPS (see Fig. S11-1) and AttractSPE™ SDB-RPS disk were sampled in four replicates. AttractSPE™ HLB (see Fig. S11-2) disks were sampled in duplicates. Each sampler is represented by one point. Substances are sorted by log *K*_OW_

With higher flow velocity, more analyte was sampled per disk. Using A-RPS disks as an example, Fig. 3 shows that the biggest increase (1.28-fold) occurred for propiconazole. Also, for the other *v*_10_ and *v*_20_ combinations and compounds, *v*_20_ data exceeded *v*_10_ data with exception of bentazon for the SDB sampler and nicosulfuron for E-RPS and HLB sampler (see Fig. S11). When all compounds were averaged within each of nine treatment combinations, *v*_20_ treatment increased uptake by about 10 to 30% (Table 2). Averaged over all nine combinations, the sampled amount per disk was 1.16 times higher in the *v*_20_ compared with *v*_10_ treatment.

**Table 2:** Mean ratios of sampled analyte amounts at different flow velocities (analyte amounts at *v*_20_ divided by analyte amounts at *v*_10_) for all three time slots and sampling phases. Right column displays the mean ratio of the three time slots for each sampling phase, respectively

Sampling phase		Time slot	*v*_20_:*v*_10_ ± SE per time slot	Mean *v*_20_:*v*_10_ ratio ± SE per sampling phase
Empore™	SDB-RPS	Days 1–7	1.1 ± 0.3	1.1 ± 0.4
		Days 8–14	1.1 ± 0.5	
		Days 1–14	1.2 ± 0.3	
AttractSPE™	SDB-RPS	Days 1–7	1.1 ± 0.3	1.2 ± 0.5
		Days 8–14	1.2 ± 0.3	
		Days 1–14	1.3 ± 0.4	
	HLB	Days 1–7	1.1 ± 0.1	1.1 ± 0.2
		Days 8–14	1.2 ± 0.2	
		Days 1–14	1.2 ± 0.2	

As expected (Vermeirssen et al. 2008), results show that uptake increases with higher flow rates as an increased flow leads to a decrease of the aqueous boundary layer and thus reduces resistance for chemical uptake. Such effects of flow on uptake are particularly evident for the sampler configuration we used, where the sampling phase is in direct contact with water without the use of a protective (diffusion-limiting) membrane. However, the use of a membrane drastically reduces uptake, particularly for the more hydrophobic compounds. So, the advantage of reduced dependency of flow is offset by a strongly reduced sampling rate (Alvarez 1999; Tran et al. 2007).

The period of increased flow velocity was relatively short compared with the duration of the experiment and also the 2-fold flow increase was relatively small. Altogether, this leads to relatively small differences in the sampled analyte amounts between *v*_10_ and *v*_20_ treatments. This is important for the interpretation of future field data. During rain events, we expect moderate flow increases at the sampling locations. Based on the results depicted in Table 2, it is expected that small differences in flow will not dramatically thwart interpretation of field data. Ideally, performance reference compounds would be available to better correct for differences in environmental conditions between sites (Booij et al. 2016; Estoppey et al. 2014). However, even after significant research efforts, a robust system is not yet available for samplers of polar organic compounds (Miège et al. 2015).

### Non-linear uptake over the 2-week sampling period

If sampling over 2 weeks either in the *v*_10_ or *v*_20_ treatment was perfectly integrative, the sum of the amounts sampled in both individual weeks should equal the amount sampled over the matching 2-week period. However, this was not the case: the sum of the individual weeks exceeded the 2-week slot for all compounds and all tested sampling phases. The relationship between amounts sampled in sub-slots and over the matching longer period can be evaluated in terms of integrative ratios (Vermeirssen et al. 2009) (see Fig. S12). These ratios ranged between 0.57 and 0.97. This indicates that either the initial sampling phase is not strictly linear and/or uptake is already curvilinear. Furthermore, the first peak sampled with the 2-week exposed samplers may have undergone a larger desorption compared with the samplers exposed for the first week only. Recent studies (Endo and Matsuura 2018; Mutzner et al. 2019) indicate that a non-linear initial uptake period can be explained by diffusion processes in porous media, i.e., the sorbent phase. To further evaluate how soon equilibrium will be reached, long-term uptake experiments are needed. However, for sampling periods of 1 to 2 weeks, there is no immediate concern about capacity limits or rapid equilibration, at least for the more hydrophobic compounds tested.

Although sorbent phases appear very similar (see also “Minor analyte uptake differences across sampling phases”), over time, differences may become apparent since the disks approach equilibrium between water and sampler as indicate partly by the integrative ratios. If equilibrium is reached, the differences in the sampled analyte masses by the different disks reflect the difference in the sampler capacities. However, the sampled analyte masses after 14 days exposure are, in most cases, very similar between the different disks (see “Minor analyte uptake differences across sampling phases”). This indicates similar sampler capacities—at least for the analyzed time frames we used.

### Sampling rates

We did not perform a full calibration study, i.e., assess uptake over time with many data points. Nevertheless, a rough estimation of sampling rates (*R*_S_) is possible by dividing the sampled mass per sampler by the water concentration. This was done for both, week 1 and week 2. The average of the quotients was divided by seven days to derive an *R*_S_ in liters per day. Although a proper calibration needs to be done for future work, this rough estimation is a useful measure to compare sampling properties between the three tested sampling phases. One has to keep in mind that desorption also effects the sampling rates, especially for compounds with no background concentration. Therefore, the analytes sampled over the short peak concentration might already have undergone desorption, which also differs with regard to the time of spiking in the two single weeks. Table 3 shows that sampling rates for the different phases range from 0.02 L day^−1^ for bentazon and Empore™ SDB-RPS at flow velocity *v*_10_ to 0.60 L day^−1^ for thiacloprid and AttractSPE™ HLB at flow velocity *v*_20_. Sampling rates shown in Table 3 reaffirm conclusions in previous discussion sections, e.g., AttractSPE™ phases have a five to six times higher sampling rate for bentazon compared with the Empore phase. Also, sampling rates for nicosulfuron are higher for the HLB phase compared with the SDB phases. All other substances have similar sampling rates for all sampling phases. Sampling rates for the AttractSPE™ HLB disks appear overall somewhat higher. However, one has to keep in mind that only duplicates have been measured for the HLB phase and these estimates are less robust than those based on the four replicates that were available for the SDB phases.

**Table 3:** Estimated sampling rates *R*_S_ for Empore™ SDB-RPS, AttractSPE™ SDB-RPS, and AttractSPE™ HLB disks for target analytes at *v*_10_ and *v*_20_ in liters per day

Substance	Empore™ SDB-RPS		AttractSPE™ SDB-RPS		AttraactSPE™ HLB	
	*v*_10_	*v*_20_	*v*_10_	*v*_20_	*v*_10_	*v*_20_
Bentazon	0.02	0.02	0.08	0.07	0.08	0.09
Nicosulfuron	0.02	0.03	0.04	0.04	0.07	0.07
Imidacloprid	0.27	0.29	0.29	0.31	0.31	0.35
Metazachlor	0.40	0.44	0.41	0.45	0.43	0.48
Thiacloprid	0.45	0.52	0.47	0.55	0.51	0.59
Flufenacet	0.43	0.50	0.44	0.51	0.47	0.54
Terbuthylazine	0.39	0.46	0.41	0.49	0.46	0.52
Propiconazole	0.18	0.21	0.18	0.26	0.23	0.26
Pendimethalin	0.17	0.22	0.17	0.22	0.19	0.22

Only few sampling rates for this combination of substances and sampling phases can be found in literature. Moreover, the sampling conditions vary which allows only an appraisal. Ahrens et al. (2015) determined sampling rates for different passive samplers including bentazon, imidacloprid, metazachlor, pendimethalin, propiconazole, and terbuthylazine. With a flow velocity of 10 cm s^−1^ at 20 °C, there were similar conditions. However, for the SDB-RPS disks, they used PES membranes. This explains the lower sampling rates by factors of 2–4 in their study. For pendimethalin, there was a factor 55 difference. This can also be explained by the PES membrane. In a previous non-published experiment, we found that the PES membrane not only slows down the uptake of pendimethalin for the SDB-RPS sampler. Moreover, the analyte remains mainly on the PES membrane when used with a SDB-RPS sampler (see Fig. S1). Also, Alvarez (1999), Tran et al. (2007), and Estoppey et al. (2019) showed that some analytes are found in high amounts associated with the PES membrane. Therefore, the use of a PES membrane may lead to false low or negative detection of certain analytes of interest that do not reach the disk itself. This is why the sampling rates found by Ahrens et al. (2015) are very low compared with the sampling rate found in this study without PES membrane.

Typically, studies attempt to correlate log *K*_OW_ with *R*_S_. Some studies found reasonable correlations (e.g., Shaw et al. 2009; Vermeirssen et al. 2013); others did not (Moschet et al. 2015). Figure 2 (also Figs. S7, S9, and S11), which shows all tested compounds ranked by log *K*_OW_, illustrates that uptake increases with log *K*_OW_, then levels and, finally, decreases for propiconazole and pendimethalin. This seems surprising and made us carefully evaluate chemical analysis data again. However, no errors could be observed. It has to be noted though that testing pendimethalin in the channel system is challenging. Due to its high log *K*_OW_ value, it sorbs strongly to the large plastic surface areas in the channels and the peak concentration did not really show up. Given *R*_S_ estimates of this study are based on two measurements only and only nine compounds were tested, we did not attempt further elucidation of relationships between compound properties and estimated sampling rates.

All *c*_w_ measurements were above LOQ (S/N ratios > 10), and this was also the case for all passive sampler extracts (S/N ratios > 10).

### Standardized sorbent materials for passive samplers

Even though 3M™ Empore™ disks became available again during the course of this study (CDS Analytical LLC, Oxford, PA, USA), our results help demonstrate to what extent it is possible to compare studies that use SDB-RPS disks of different producers. Knowledge about the comparability of sampling phases from different producers as well as comparisons between different sampling phases is of great importance for future harmonization and standardization of passive sampling. Currently, many sampler types exist (e.g., POCIS, Chemcatcher, oDGT) which can be operated in different configurations (e.g., sampler housing, size, membrane and sorbent type). Although each type may have its benefits, method standardization will be advantageous to support regulatory acceptance and implementation of passive sampling. Comparative data on sorbent performance can further help to transfer methods and products for other concentration and purification methods that employ SDB-RPS disks (e.g., SPE (Charles et al. 2001, Richter and Oertel 1999)). Moreover, knowledge on comparability enables faster switches from one producer to another in case of another halt in future production. This is of special importance as long as there is no standardized material for passive sampling with guaranteed availability in the near future. Discontinuation of passive sampler material is of major concern since this might halt the ongoing improvement and implementation of passive sampling techniques. Moreover, there could be other reasons to switch the product such as price differences between different producers.

Presented results show that AttractSPE™ SDB-RPS is a well-suited sampling phase for the tested analytes and has similar sampling properties as Empore SDB-RPS. Indeed, along the same lines, it would be of great interest to test AttractSPE™ HLB disks versus the established HLB disks from Biotage. Additional comparison studies between different sampling phases and producers would increase the understanding of comparability of monitoring studies (if sampler phase producers were different). Such comparative work might also identify alternative products and would indicate whether, next to the sampling phase, properties such as carrier material and polymer loading as a percentage of the disk might be of importance for some analyte groups.

## Conclusion

The results of this study show a good comparability of the three tested sampling phases. There are only minor differences in their sampling properties with nicosulfuron being sampled more by the AttractSPE™ HLB phase and bentazon being sampled more by the products from Affinisep. However, all selected analytes can be sampled by all three sampling phases. No difficulties for the limits of quantification with either of these samplers have been encountered. Overall, the sampling rates for the HLB receiving phase seem to be somewhat higher compared with the SDB-RPS phases. Due to the focus on SDB-RPS phases (four replicates), this can however not be proven statistically in this study. Since there are some analytes like bentazon and nicosulfuron, which show relevant differences in the sampling properties between the different receiving phases, the suitability of a sampling phase for the compounds of interest needs to be verified in advance.

## Supplementary information

ESM 1 (DOCX 1722 kb)

## Data Availability

The datasets used and/or analyzed during the current study are available from the corresponding author on reasonable request.
