# Supplementary material for: Passive sampler phases for pesticides: evaluation of AttractSPE™ SDB-RPS and HLB versus Empore™ SDB-RPS
Source: Environ Sci Pollut Res Int. 2021 Jan 12;28(9):11697–707. doi: 10.1007/s11356-020-12109-9 (PMC7886826; doi:10.1007/s11356-020-12109-9)
Supplement: Supplementary file 1 — (DOCX 1722 kb) [file 11356_2020_12109_MOESM1_ESM.docx]

# Supporting Information

***Article:***

*Passive sampler phases for pesticides: Evaluation of – AttractSPE^TM^ SDB-RPS and HLB versus Empore^TM^ SDB-RPS*

***Journal:***

*Environmental Science and Pollution Research*

***Authors:***

*Benjamin Becker*, Christian Kochleus, Denise Spira, Christel Möhlenkamp, Julia Bachtin, Stefan Meinecke, Etiënne L. M. Vermeirssen*

***Corresponding author (*):***

*Benjamin.becker@bafg.de*

*German Federal Institute of Hydrology (BfG), Am Mainzer Tor 1, 56068 Koblenz, Germany*

## S1 Comparison of analyte uptake in SDB disks with and without PES membrane

Fig. S1-1 Batch assay in a 1 L beaker spiked with 9 substances with an absolute concentration of 66 ng for each analyte. Three Empore^TM^ SDB-RPS disks without membrane were added to the beaker for 14 days. Afterwards, the disks were extracted and analyzed by HPLC-MS/MS. At the time this experiment was conducted, Thiacloprid was not chosen as analyte, yet, and, therefore, is not shown here. The dotted line indicates the theoretical maximum concentration.

Fig. S1-2 Batch assay in a 1 L beaker spiked with 12 substances with an absolute concentration of 66ng for each analyte. Three Empore^TM^ SDB-RPS disks with PES-membrane were added to the beaker for 14 days. Disks and membranes were separately analyzed, results for both are displayed in the graph. The dashed line indicates the theoretical maximum amount.

## S2 SST housing for SDB-RPS disks


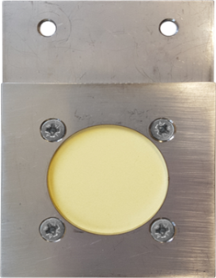


Fig. S2 Custom stainless steel housing for the exposure of the SDB disks with a one-sided opening (Ø = 40 mm). Since the experiences made with these types of sampler housings in (Vermeirssen et al. 2012) were very good, SST housings were also used in this study instead of commercially available Chemcatchers® (picture: BfG).

## S3 Picture of the stream channel with the holders for SST housings


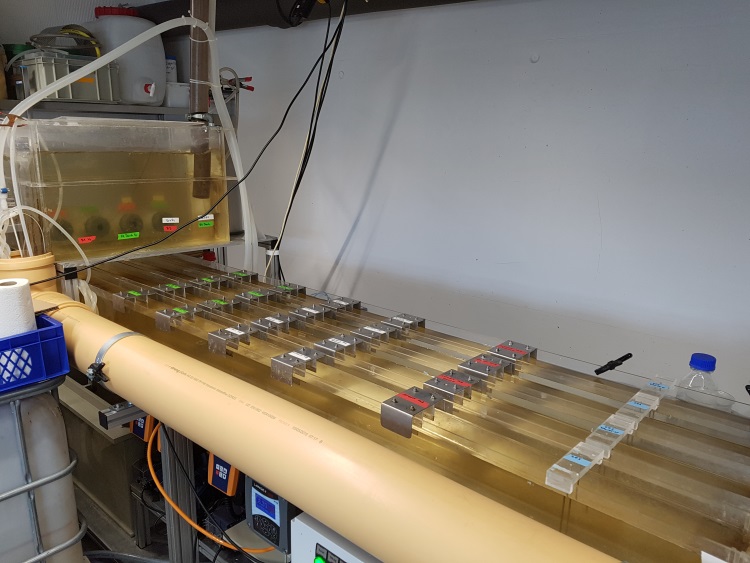


Fig. S3 Stream channel system of the Swiss Centre for Applied Ecotoxicology which was used in this study. Five metal holders for stainless steel passive sampler housings can be seen for each channel. Green labeld holders carry Empore^TM^ SDB-RPS disks, white labels carry AttractSPE^TM^ SDB-RPS disks and red labels carry AttractSPE^TM^ HLB disks. Blue labeled acrylic glass holders were used for silicone rubber sheets for further experiments (picture: BfG).

## S4 Temperature and pH development

**Fig. S4** Temperature and pH values over the test duration of two weeks. Data were recorded using HOBO UA-002-08 8K Pendant® (Onset Computer Corporation, Bourne, MA, USA) temperature sensor and pHD-s SC (Hach Lange GmbH, Düsseldorf, Germany) pH logger.

## S5 Analyte concentration in water over time of the experiment


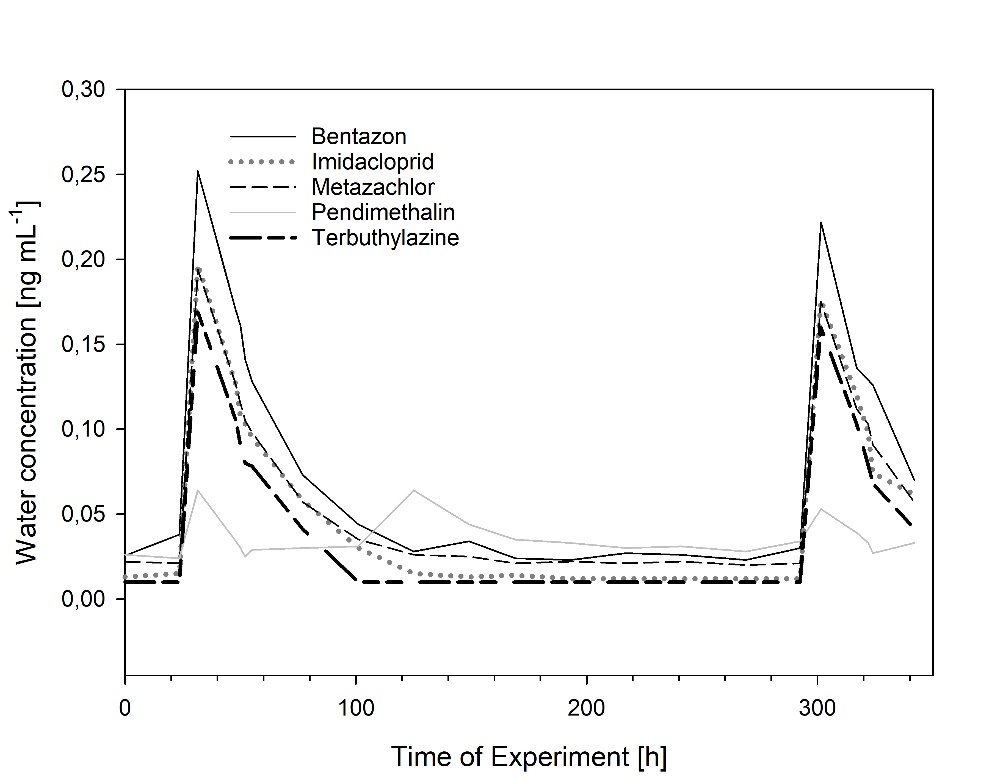


**Fig. S5-1** Water concentration in ng mL^-1^ over the time of the experiment for Bentazon, Imidacloprid, Metazachlor, Pendimethalin and Terbuthylazine.


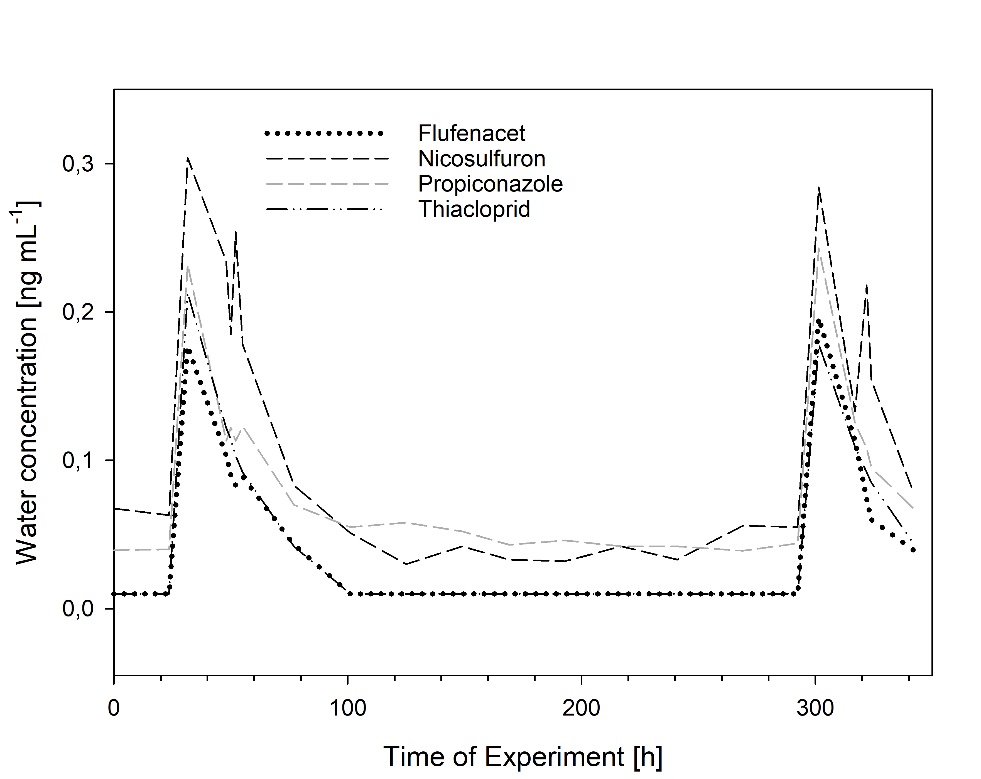


**Fig. S5-2** Water concentration in ng mL^-1^ over the time of the experiment for Flufenacet, Nicosulfuron, Propiconazol and Thiacloprid.

## S6 Specifications for HPLC-MS/MS analysis including main settings for analysis as well as limit of detection and limit of quantification

**Tab. S6-1** Specifications of HPLC-MS/MS analysis in ESI positive and ESI negative mode. Given are two ion transitions for each analyte, declustering potential (DP), Entrance potential (EP), collision energy (CE) and cell exit potential (CXP).

| **ESI pos.** | **Analyte** | ***Q1 Mass (Da)*** | ***Q2 Mass (Da)*** | ***ID*** | ***DP*** | ***EP*** | ***CE*** | ***CXP*** |
| --- | --- | --- | --- | --- | --- | --- | --- | --- |
|  | Flufenacet | 364.0 | 194.0 | Flufenacet _1 | 51 | 10 | 15 | 6 |
|  |  | 364.0 | 152.1 | Flufenacet_ 2 | 51 | 10 | 25 | 10 |
|  | Flufenacet-d4 | 368.0 | 198.0 | Flufenacet-d4_1 | 61 | 10 | 15 | 8 |
|  |  | 368.0 | 155.9 | Flufenacet-d4_2 | 61 | 10 | 23 | 10 |
|  | Imidacloprid | 256.0 | 175.1 | Imidacloprid _1 | 71 | 10 | 25 | 8 |
|  |  | 256.0 | 208.8 | Imidacloprid_ 2 | 71 | 10 | 19 | 18 |
|  | Imidacloprid-d4 | 260.0 | 179.1 | Imidacloprid-d4_ 1 | 71 | 10 | 25 | 8 |
|  |  | 260.0 | 212.8 | Imidacloprid-d4_ 2 | 71 | 10 | 19 | 18 |
|  | Metazachlor | 278.0 | 134.0 | Metazachlor_1 | 36 | 10 | 27 | 6 |
|  |  | 278.0 | 209.9 | Metazachlor_2 | 36 | 10 | 13 | 8 |
|  | Metazachlor-d6 | 284.0 | 140.2 | Metazachlor-d6 _1 | 31 | 10 | 27 | 12 |
|  |  | 284.0 | 216.1 | Metazachlor-d6_2 | 31 | 10 | 15 | 16 |
|  | Nicosulfuron | 411.0 | 181.9 | Nicosulfuron_1 | 86 | 10 | 27 | 8 |
|  |  | 411.0 | 212.9 | Nicosulfuron_2 | 86 | 10 | 23 | 10 |
|  | Nicosulfuron-d6 | 417.1 | 218.8 | Nicosulfuron-d6_2 | 91 | 10 | 23 | 18 |
|  |  | 417.1 | 181.8 | Nicosulfuron-d6-1 | 91 | 10 | 25 | 12 |
|  | Pendimethalin | 282.1 | 211.9 | Pendimethalin_1 | 36 | 10 | 13 | 8 |
|  |  | 282.1 | 193.9 | Pendimethalin_2 | 36 | 10 | 23 | 8 |
|  | Pendimethalin-d5 | 287.0 | 213.2 | Pendimethalin-d5_1 | 16 | 10 | 15 | 14 |
|  |  | 287.0 | 194.1 | Pendimethalin-d5_2 | 16 | 10 | 25 | 10 |
|  | Propiconazole | 342.0 | 158.8 | Propiconazole_1 | 86 | 10 | 33 | 10 |
|  |  | 342.0 | 122.8 | Propiconazole_2 | 86 | 10 | 81 | 10 |
|  | Propiconazole-d3 | 345.0 | 162.0 | Propiconazole-d3_1 | 91 | 10 | 33 | 12 |
|  |  | 347.0 | 164.0 | Propiconazole-d3_2 | 91 | 10 | 23 | 20 |
|  | Terbuthylazine | 230.0 | 173.9 | Terbuthylazine_1 | 81 | 10 | 21 | 12 |
|  |  | 230.0 | 104.0 | Terbuthylazine_2 | 81 | 10 | 31 | 7 |
|  | Terbuthylazine-d5 | 235.0 | 179.0 | Terbuthylazine-d5_1 | 76 | 10 | 21 | 2 |
|  |  | 235.0 | 100.9 | Terbuthylazine-d5_2 | 76 | 10 | 33 | 26 |
|  | Thiacloprid | 252.9 | 125.9 | Thiacloprid_1 | 86 | 10 | 25 | 5 |
|  |  | 252.9 | 90.0 | Thiacloprid_2 | 86 | 10 | 53 | 8 |
|  | Thiacloprid-d4 | 256.9 | 126.0 | Thiacloprid-d4_1 | 56 | 10 | 27 | 4 |
|  |  | 256.9 | 89.9 | Thiacloprid-d4_2 | 56 | 10 | 45 | 10 |
| **ESI neg.** | **Analyte** | ***Q1 Mass (Da)*** | ***Q2 Mass (Da)*** | ***ID*** | ***DP*** | ***EP*** | ***CE*** | ***CXP*** |
|  | Bentazon | 238.8 | 131.9 | Bentazon 1 | -70 | -10 | -34 | -9 |
|  |  | 238.8 | 196.8 | Bentazon 2 | -70 | -10 | -28 | -15 |
|  | Bentazon-d7 | 245.9 | 132.0 | Bentazon-d7_1 | -65 | -10 | -32 | -7 |
|  |  | 245.9 | 182.0 | Bentazon-d7_2 | -65 | -10 | -26 | -17 |
|  | Metazachlor ESA | 322.0 | 120.8 | Metazachlor ESA_1 | -95 | -10 | -28 | -9 |
|  |  | 322.0 | 148.0 | Metazachlor ESA_2 | -95 | -10 | -32 | -11 |

**Tab. S6-2** Limit of detection (LOD) and limit of quantification (LOQ) in ng disk-1 for passive sampler and in ng mL-1 for water samples.

| Analyte | LOD [ng disk^-1^] | LOQ [ng disk^-1^] | LOD [ng mL^-1^] | LOQ [ng mL^-1^] |
| --- | --- | --- | --- | --- |
| Flufenacet | 0.1 | 0.4 | 0.003 | 0.01 |
| Imidacloprid | 1.2 | 4.0 | 0.030 | 0.10 |
| Metazachlor | 0.1 | 0.4 | 0.003 | 0.01 |
| Nicosulfuron | 1.2 | 4.0 | 0.015 | 0.05 |
| Pendimethalin | 1.2 | 4.0 | 0.003 | 0.01 |
| Propiconazole | 0.1 | 0.4 | 0.003 | 0.01 |
| Terbuthylazine | 0.1 | 0.4 | 0.003 | 0.01 |
| Thiacloprid | 0.1 | 0.4 | 0.003 | 0.01 |
| Bentazon | 0.2 | 0.8 | 0.006 | 0.02 |
| Metazachlor ESA | 0.6 | 2.0 | 0.006 | 0.02 |

## S7 Analyte uptake for each individual week at v_20_


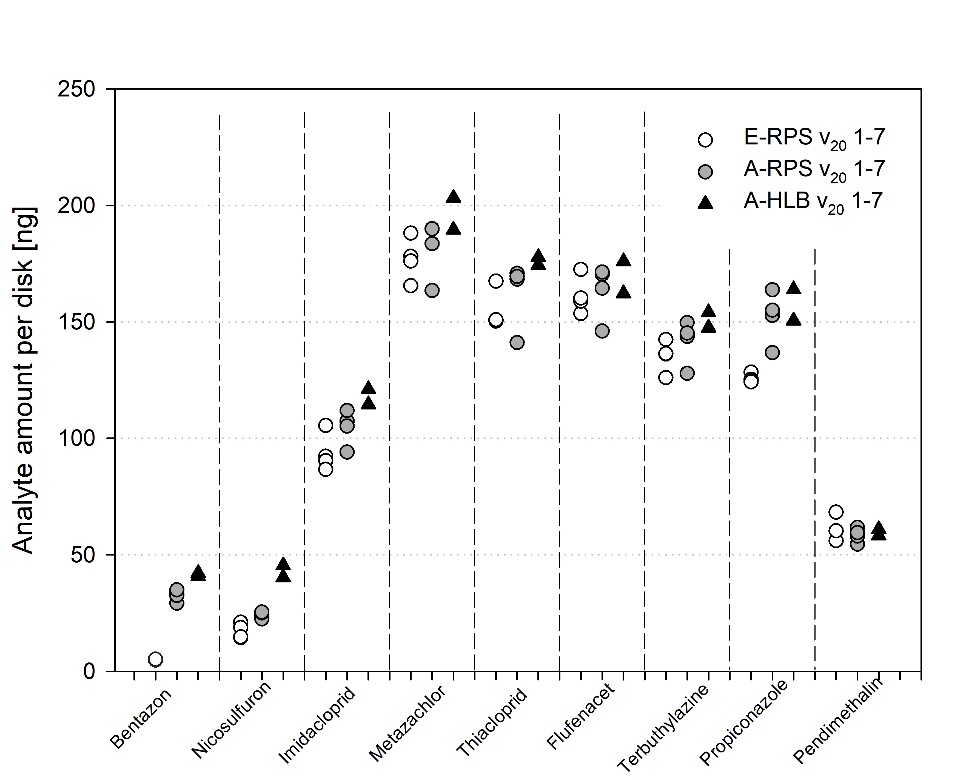


Fig. S6-1 Analyte concentrations in ng disk^-1^ after the first week of exposure (day 1-7) at v_20_ in the stream channel spiked with nine compounds (substances are sorted by log K_OW_). Empore^TM^ SDB-RPS and AttractSPE^TM^ SDB-RPS disks were sampled in four replicates. AttractSPE^TM^ HLB disks were sampled in duplicates.


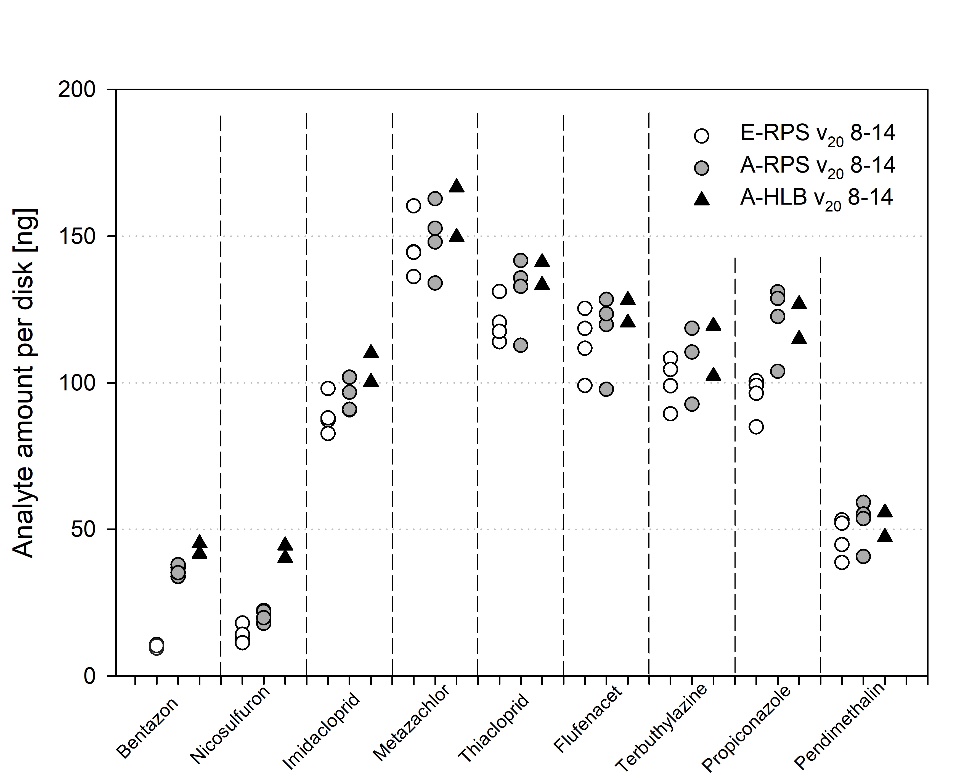


Fig. S6-2 Analyte concentrations in ng disk^-1^ the second week of exposure (day 8-14) at v_20_ in the stream channel spiked with nine compounds (substances are sorted by log K_OW_). Empore^TM^ SDB-RPS and AttractSPE^TM^ SDB-RPS disks were sampled in four replicates. AttractSPE^TM^ HLB disks were sampled in duplicates.

## S8 Comparison of Empore^TM^ SDB-RPS and AttractSPE^TM^ SDB-RPS disks

For the comparison of the two SDB-RPS phases four replicates per analyte for v_10_ and v_20_ each were compared.


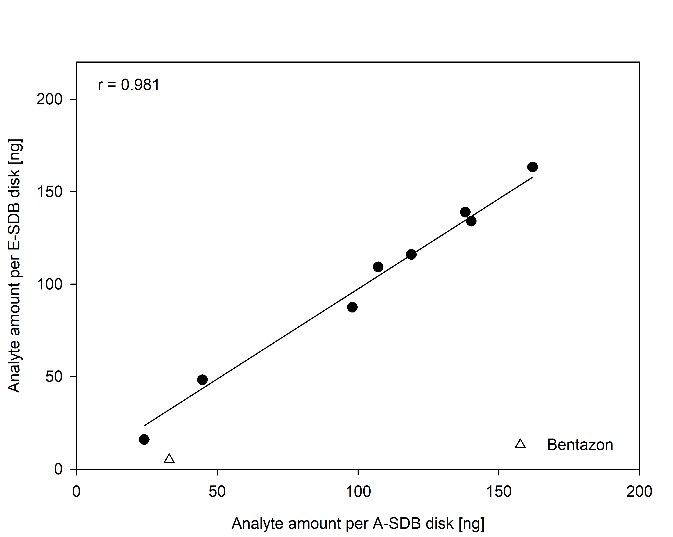


Fig. S8-1 Correlation of Empore^TM^ SDB-RPS disks vs AttractSPE^TM^ SDB-RPS disks for the sampled mass in ng disk^-1^ after week 1 at flow velocity v_10_. Except for Bentazon, there is a high correlation between both samplers with r = 0.981.

| **A** | **B** |
| --- | --- |
| 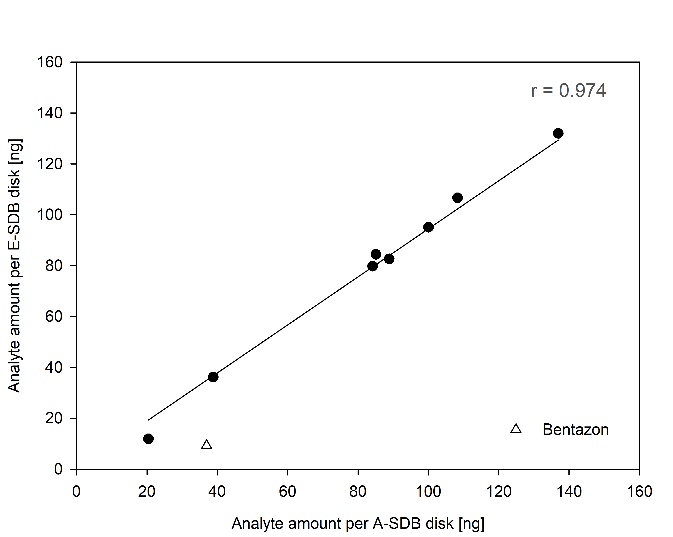 | 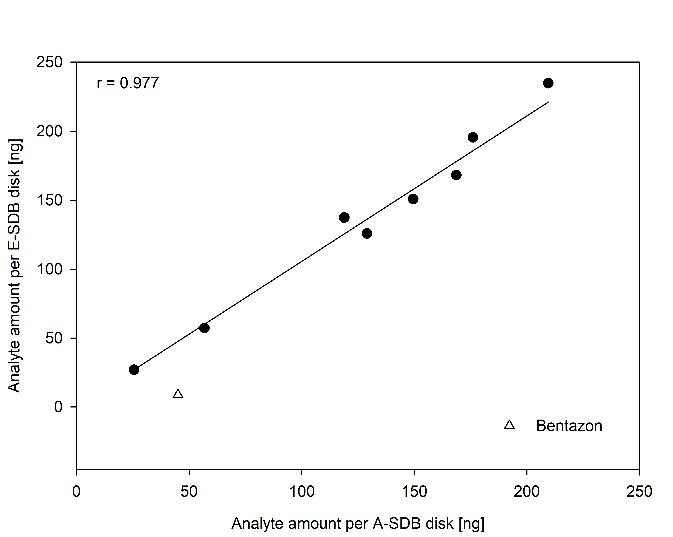 |

Fig. S8-2 Correlation of Empore^TM^ SDB-RPS disks vs AttractSPE^TM^ SDB-RPS disks for the sampled mass in ng disk^-1^ after week 2 at flow velocity v_10_ (A) and after 14 d of exposure at flow velocity v_10_ (B). With r = 0.974 during the second week (A) and r = 0.977 after 14 d exposure at flow velocity v_10_ (B) the assumption was reaffirmed, again, except for Bentazon.

| **A** | **B** |
| --- | --- |
| 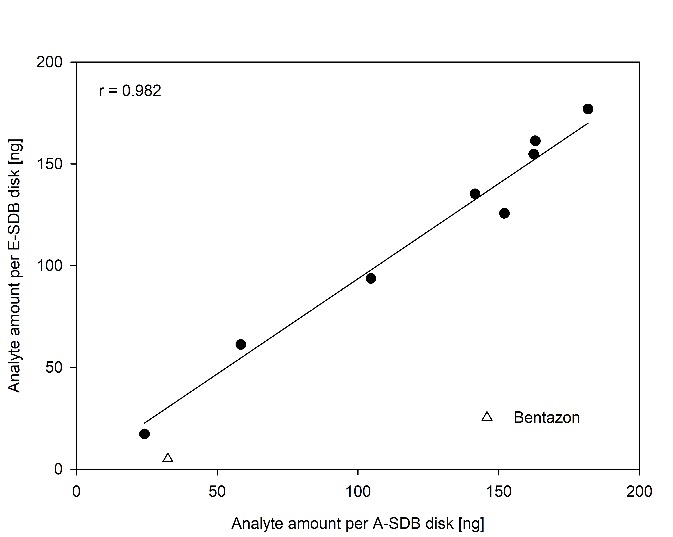 | 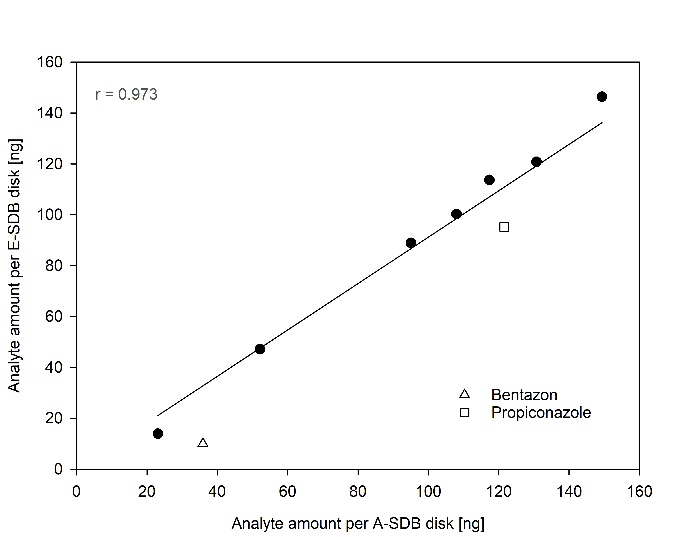 |

Fig. S8-3 Correlation of Empore^TM^ SDB-RPS disks vs AttractSPE^TM^ SDB-RPS disks for the sampled mass in ng disk^-1^ after week 1 (A) at flow velocity v_20_ and for the second week at flow velocity v_20_ (B).


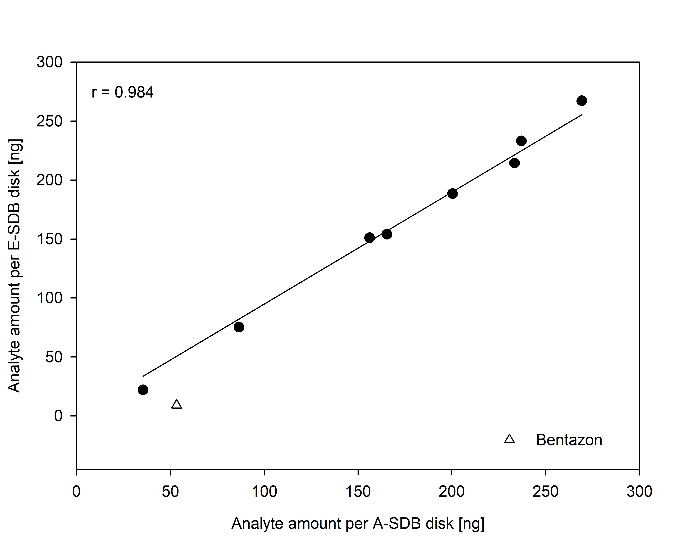


Fig. S8-4 Correlation of Empore^TM^ SDB-RPS disks vs AttractSPE^TM^ SDB-RPS disks for the sampled mass in ng disk^-1^ after 14 d exposure at flow velocity v_20_.

The v_20_ results are similar: with r ≥0.9, these results show that the samplers with the same sampling phase from the two different producers have very similar sampling properties with exception of Bentazon and Propiconazole for the second week.

With r = 0.982 for the first week (figure S8-3 A) there is a high correlation between both samplers and r = 0.973 (S8-3 B) during the second week and a r = 0.984 after 14 d exposure at flow velocity v_20_ (S8-4) reaffirm this assumption.

## S9 Analyte uptake for all exposure scenarios at v_10_


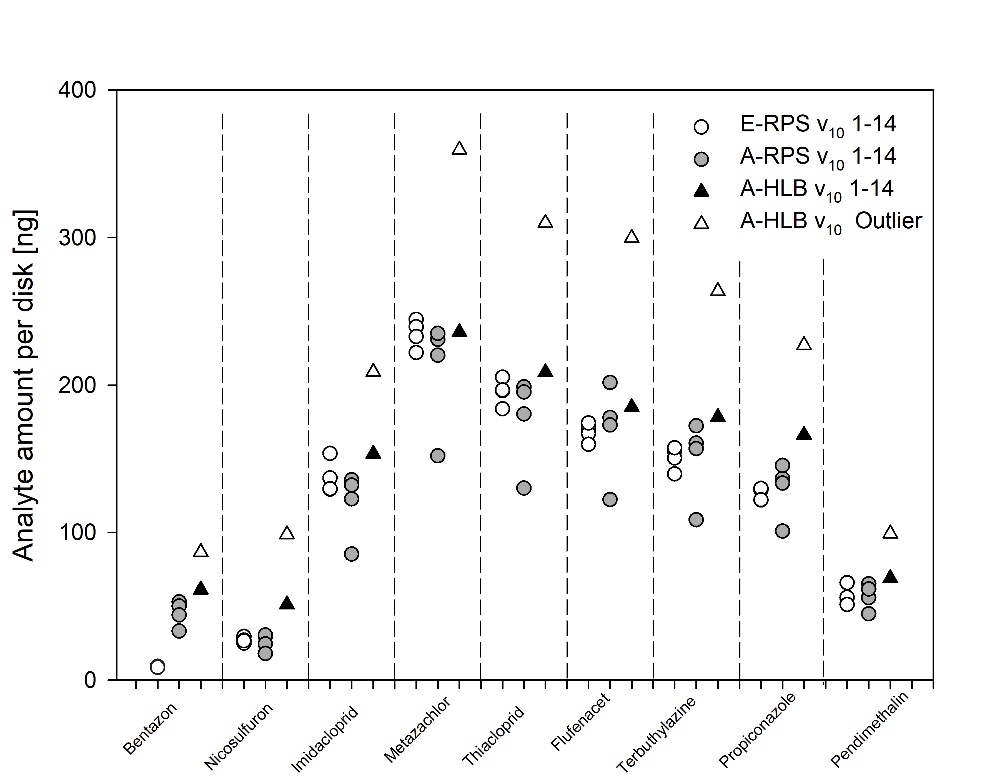


Fig. S9-1 Analyte concentrations in ng disk^-1^ after 14 d of exposure at v_10_ in the stream channel spiked with nine compounds (substances are sorted by log K_OW_). Empore^TM^ SDB-RPS and AttractSPE^TM^ SDB-RPS disks were sampled in four replicates. AttractSPE^TM^ HLB disks were sampled in duplicates.

Very high amounts of all analytes were determined for one AttractSPE^TM^ HLB sampler at v_10_ after 14 d. Careful evaluation of the data indicates that not enough internal standard was spiked to this sample. The peak sizes of the analytes are similar in terms of height and area; however, all the internal standard peaks are smaller. Thus, the IS corrected analyte concentration increases. It is, however, unlikely that this replicate has a higher matrix background (same exposure as other disk, unlikely that suppression is the same for all peaks coming at different retention time) or poorer extraction efficiency (analyte peaks are the same as for the other compounds). The only remaining explanation is that inadvertently, less internal standard was added to this sample. For this reason, the replicate with higher amounts per disk is shown with a different symbol and was not considered for data interpretation.


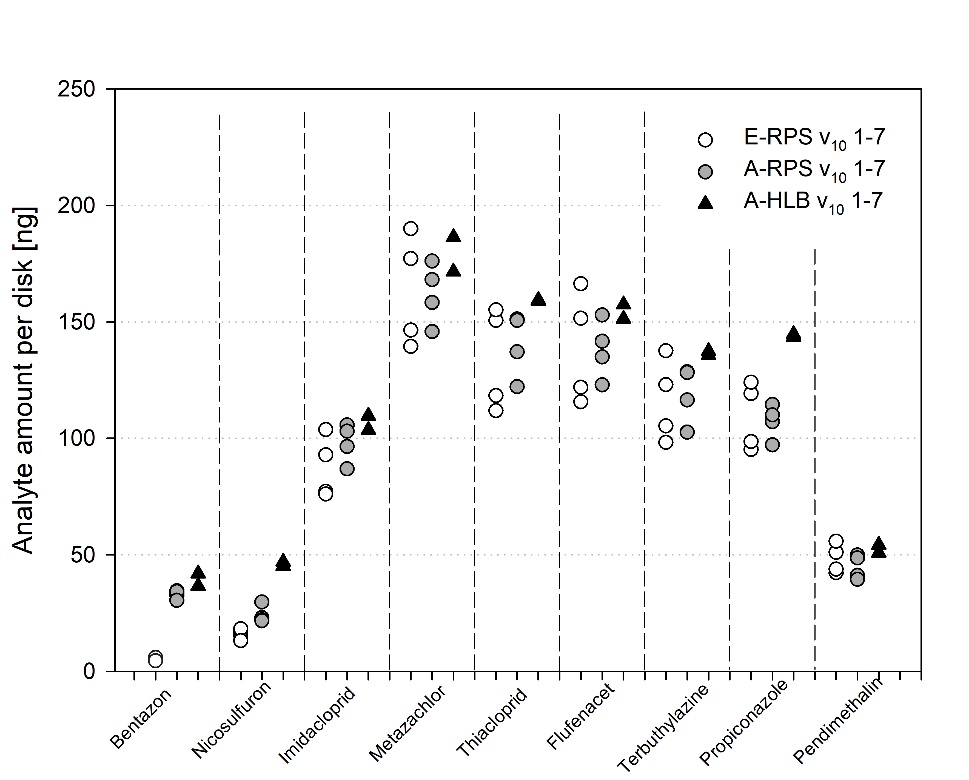


Fig. S9-2 Analyte concentrations in ng disk^-1^ after the first week of exposure (day 1-7) at v_10_ in the stream channel spiked with nine compounds (substances are sorted by log K_OW_). Empore^TM^ SDB-RPS and AttractSPE^TM^ SDB-RPS disks were sampled in four replicates. AttractSPE^TM^ HLB disks were sampled in duplicates.


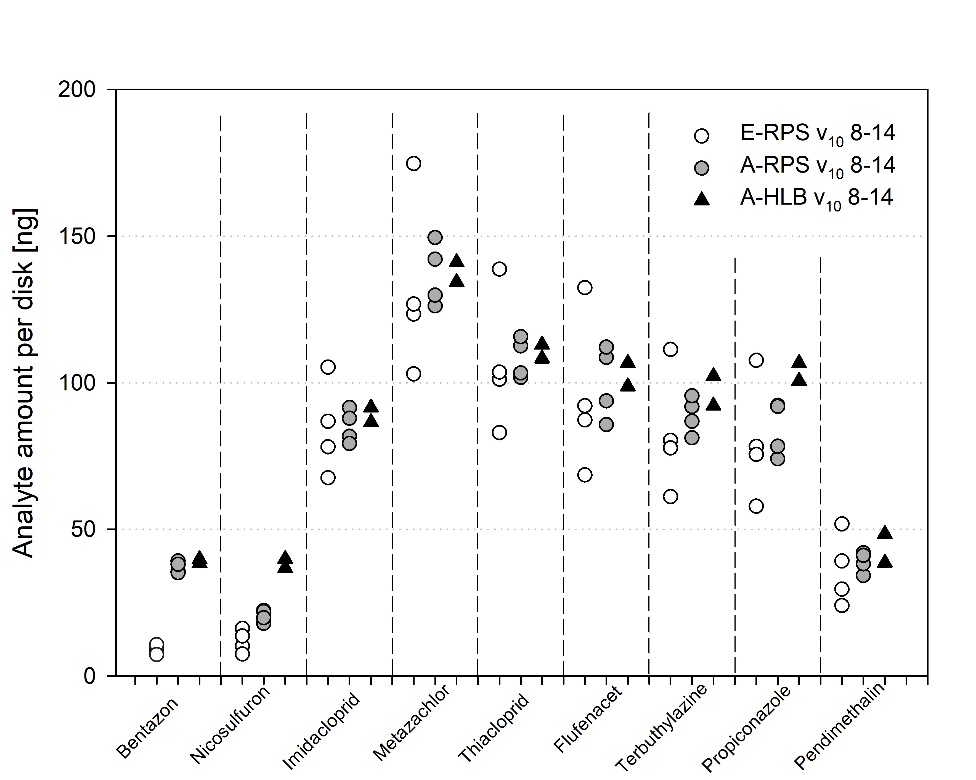


Fig. S9-3 Analyte concentrations in ng disk^-1^ the second week of exposure (day 8-14) at v_10_ in the stream channel spiked with nine compounds (substances are sorted by log K_OW_). Empore^TM^ SDB-RPS and AttractSPE^TM^ SDB-RPS disks were sampled in four replicates. AttractSPE^TM^ HLB disks were sampled in duplicates.

## S10 Comparison of AttractSPE^TM^ SDB-RPS and AttractSPE^TM^ HLB disks

Amounts of the analysed substances sampled by AttractSPE™ SDB-RPS and HLB disks were highly correlated, as can be seen in S10 (A) for v_20_ for the first week and for two weeks respectively in S10 (B). The slopes of the linear regressions (7 d: 0.985; 14 d: 0.970) are close to 1 showing the comparability of the two phases with slightly higher amounts sampled by HLB disks for the tested flow rates and sampling periods. One exception is Nicosulfuron which is sampled by a factor of approximately two better with HLB samplers under all conditions. For v_10_ the correlation shows the same outcome.

| **A** | **B** |
| --- | --- |
| 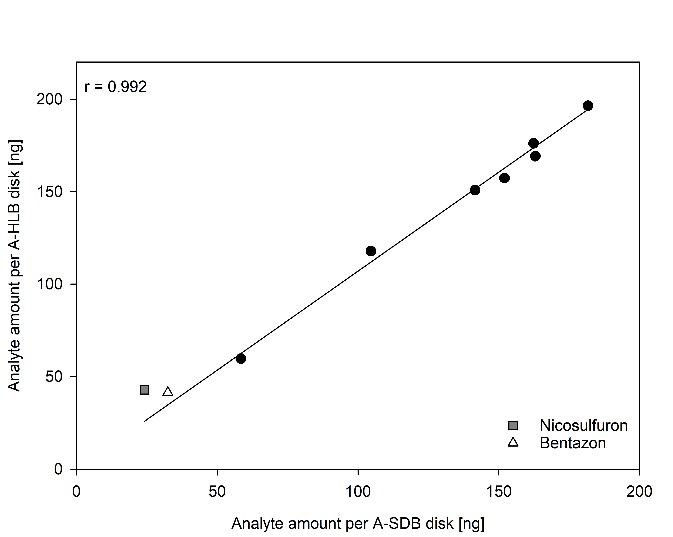 | 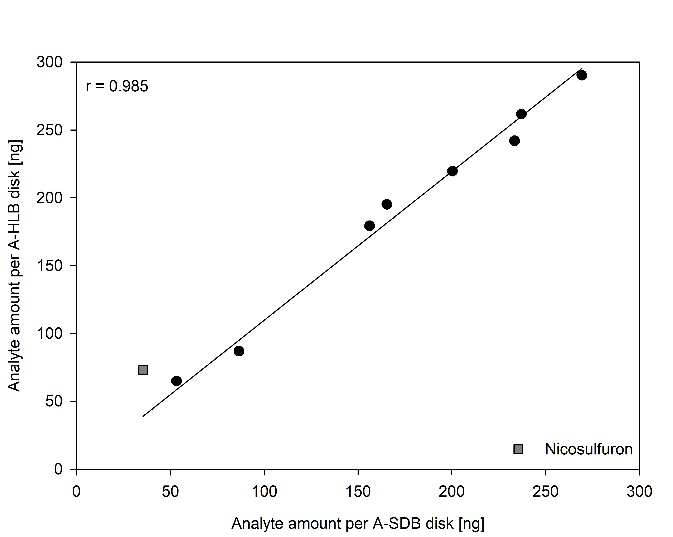 |

**Fig. S10** Correlation of sampled analyte masses of the two AttractSPE^TM^ samplers in ng disk^-1^. Plotted is the mass per AttractSPE^TM^ SDB-RPS disk vs mass per AttractSPE^TM^ HLB disk after one week (A) with r = 0.992 and after two weeks (B) with r = 0.985 for flow velocity v_20_. AttractSPE^TM^ SDB-RPS samplers were conducted with four replicates (n = 4) and AttractSPE^TM^ HLB samplers were conducted in duplicates (n = 2).

## S11 Comparison of analyte exposure after 14 days for v_10_ vs v_20_


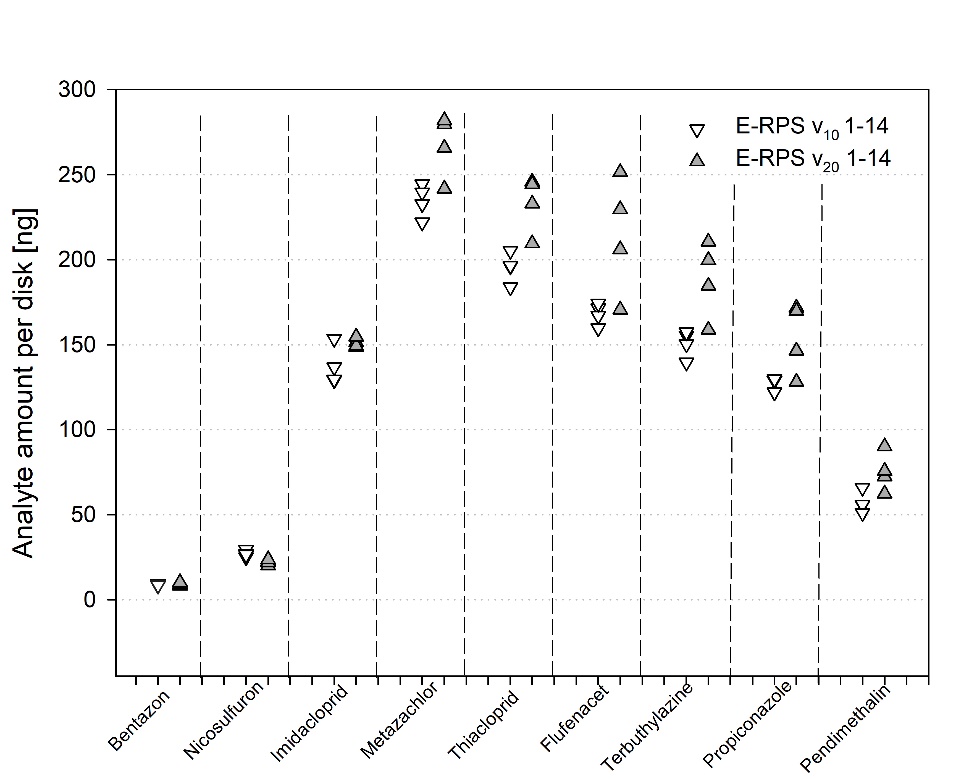


Fig. S11-1 Analyte concentrations in ng disk^-1^ for the Empore^TM^ SDB-RPS disks after 14 d of exposure in the stream channel at v_10_ (▽) and at v_20_ (▲). Bentazon, Metazachlor and Nicosulfuron were added as a continuous background concentration of 21.5 ng L^-1^. Additionally, a 215 ng L^-1^ peak of all analytes was applied for 8 h on day 2 and day 12. Empore^TM^ SDB-RPS disk were sampled in four replicates. Each sampler is represented by one symbol. Substances are sorted by log K_OW_.


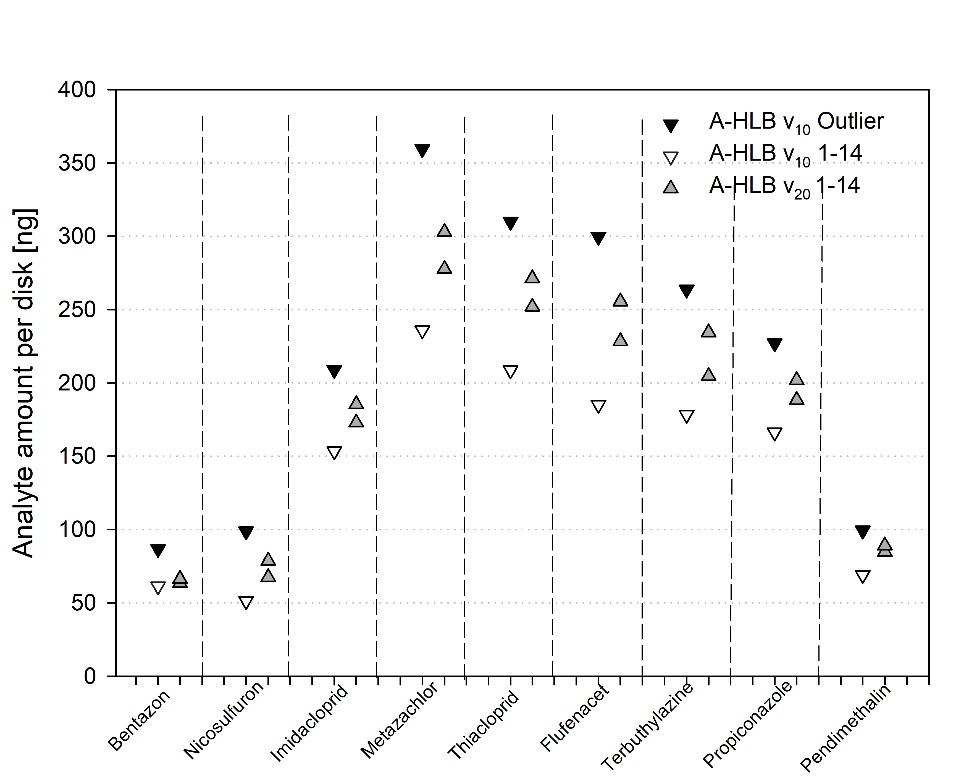


Fig. S11-2 Analyte concentrations in ng disk^-1^ for the AttractSPE^TM^ HLB disks after 14 d of exposure in the stream channel at v_10_ (▽) and at v_20_ (▲). Bentazon, Metazachlor and Nicosulfuron were added as a continuous background concentration of 21.5 ng L^-1^. Additionally, a 215 ng L^-1^ peak of all analytes was applied for 8 h on day 2 and day 12. AttractSPE^TM^ HLB disk were sampled in duplicates. Each sampler is represented by one symbol. Substances are sorted by log K_OW_.

For the HLB exposure at v_10_ the higher concentration was marked as outlier as explained in S9.

## S12 Integrative ratios for tested analytes

**Tab. S12** For all three sampling phases, integrative sampling ratios (sampled mass after 14 d over sum of sampled masses of day 1-7 and day 8-14) were calculated for the tested analytes. For perfectly integrative samplers, this ratio would be 1. In this study, values between 0.57 and 0.97 were obtained.

|  | **v_10_** | | |  | **v_20_** | | |
| --- | --- | --- | --- | --- | --- | --- | --- |
|  | E-RPS | A-RPS | A-HLB |  | E-RPS | A-RPS | A-HLB |
| **Bentazon** | 0.61 | 0.65 | 0.94 |  | 0.62 | 0.78 | 0.76 |
| **Flufenacet** | 0.72 | 0.71 | 0.94 |  | 0.78 | 0.83 | 0.82 |
| **Metazachlor** | 0.80 | 0.70 | 0.94 |  | 0.83 | 0.81 | 0.82 |
| **Nicosulfuron** | 0.97 | 0.57 | 0.89 |  | 0.71 | 0.75 | 0.86 |
| **Pendimethalin** | 0.68 | 0.68 | 0.88 |  | 0.69 | 0.78 | 0.78 |
| **Terbuthylazine** | 0.76 | 0.72 | 0.95 |  | 0.80 | 0.80 | 0.84 |
| **Propiconazole** | 0.67 | 0.67 | 0.79 |  | 0.70 | 0.61 | 0.70 |
| **Imidacloprid** | 0.80 | 0.65 | 0.93 |  | 0.83 | 0.78 | 0.80 |
| **Thiacloprid** | 0.81 | 0.71 | 0.96 |  | 0.85 | 0.81 | 0.84 |
